# Bridging the Gap Between Remote Sensing and Plant Phenotyping—Challenges and Opportunities for the Next Generation of Sustainable Agriculture

**DOI:** 10.3389/fpls.2021.749374

**Published:** 2021-10-22

**Authors:** Miriam Machwitz, Roland Pieruschka, Katja Berger, Martin Schlerf, Helge Aasen, Sven Fahrner, Jose Jiménez-Berni, Frédéric Baret, Uwe Rascher

**Affiliations:** ^1^Department of Environmental Research and Innovation, Luxembourg Institute of Science and Technology, Belval, Luxembourg; ^2^Institute of Bio and Geosciences, Plant Sciences, Forschungszentrum Jülich, Helmholtz-Verband Deutscher Forschungszentren, Jülich, Germany; ^3^Department of Geography, Ludwig-Maximilians-Universität München, Munich, Germany; ^4^Department of Environmental Systems Science, Crop Science, Eidgenössische Technische Hochschule (ETH) Zurich, Zurich, Switzerland; ^5^Instituto de Agricultura Sostenible, Consejo Superior de Investigaciones Científicas, Cordoba, Spain; ^6^INRAE-EMMAH-CAPTE, Avignon, France; ^7^Forschungszentrum Jülich, Institute of Bio- and Geosciences Plant Sciences (IBG-2), Jülich, Germany

**Keywords:** remote sensing, high-throughput field phenotyping, unmanned aerial vehicles (UAVs), multi-sensor synergies, open-data standards, vegetation traits, radiative transfer models (RTM), smart farming

## Introduction

Sustainable and resilient agriculture with a low impact on the environment is pivotal to ensure food security for a growing global population. This is of particular importance faced with the unprecedented challenge of climate change (FAO., 2017) for crop production. Sustainable intensification or currently rather the conservation of yield (Rosenqvist et al., 2019) requires the consideration of the entire crop production pipeline, ranging from breeding and identifying varieties adapted to specific environmental conditions, to improving agricultural land management (agriculture 5.0, Saiz-Rubio and Rovira-Más, 2020). An essential aspect of these efforts is the quantitative assessment of the plant traits contributing to increased, reliable production and the efficient use of resources, such as nutrients or water. Faced climate change and the appearance of more frequent and intense stress events, there is a need for resilient breeding lines, as summarized in the review of Razzaq et al. (2021). Besides drought stress, heat stress is expected to have a major negative impact on yield in Europe (Semenov and Shewry, 2011).

In this context, two areas of research, plant phenotyping and remote sensing, are becoming increasingly important. Field phenotyping refers to a quantitative description of a plant's phenotype—i.e., its anatomical, ontogenetical, physiological, and biochemical properties—in its natural environment (Walter et al., 2017). Remote sensing in the agricultural context is the observation of vegetation by a remote device and the retrieval of its qualitative or quantitative properties. While remote sensing and plant phenotyping researchers are both interested in the interaction of plant growth with the environment (including management practices), the two fields have a different focus. Traditionally, remote sensing is used to estimate spatial trends across the landscape, while plant phenotyping aims to remove spatial effects in their data in order to investigate the genetic effects of different plant varieties in response to the prevailing environmental conditions. Nevertheless, both disciplines are united in their efforts to estimate plant traits and explain apparent differences in the phenotype precisely (Aasen and Herrera, (under review)).

**Figure 1:**
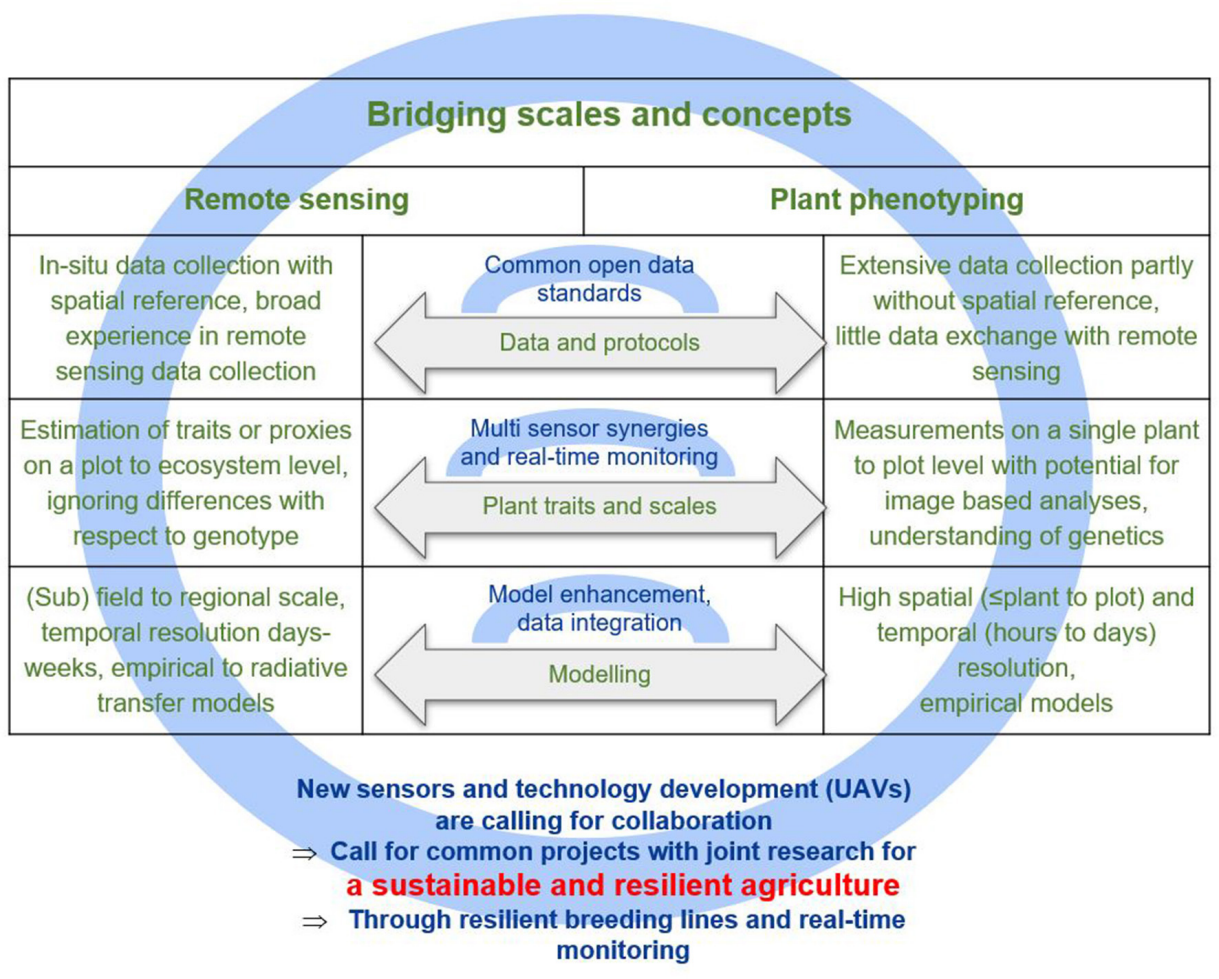
A closer collaboration of remote sensing and plant phenotyping has the potential to foster research for a sustainable and resilient agriculture. Common topics between the two communities (gray arrows) have been identified for collaboration and cutting-edge research. In the left and right columns, differences between remote sensing and plant phenotyping are listed regarding the three topics. In blue, the potential for future collaboration is indicated.

Driven by the need for new concepts in sustainable agriculture, an increased use of remote sensing approaches in field phenotyping and vice versa has been observed over the last decade. On one hand, field phenotyping has increasingly deployed imaging instruments traditionally used in remote sensing (Johansen et al., 2019) to meet the need for increased throughput in field phenotyping (Araus and Cairns, 2014). The analysis of remote sensing data by non-experts without full knowledge of the sensing principles hampers the exploitation of the full potential of the methods at hand. On the other hand, remote sensing scientists have started to estimate plant traits and analyze data from breeding experiments (Yang et al., 2017). However, their findings are often not interpreted in light of the physiological processes that shape the relation of a crop within the environment. Additionally, there are differences in input data, acquisition protocols, plant trait definitions, and retrieval models that hinder close cooperation between the two disciplines (Figure 1).

Facilitating exchange between the two disciplines offers possibilities to trigger cross-fertilization: An improved understanding of the target traits will allow the remote sensing community to develop more precise and ultimately more useful tools. Likewise, establishing state-of-the-art remote sensing methods as plant phenotyping tools will allow an improved understanding and modeling of crops in dynamic environments. Ultimately, this exchange has the potential to stimulate growth in both communities and their interconnection may lead to new developments toward more sustainable agriculture. There is a need for multiple stress-resilient breeding lines combined with a need for multi-site and multi-regional testing (Rosenqvist et al., 2019). Breeders and plant phenotyping need to provide breeding lines that are able to cope with unprecedented stress conditions. To target sustainable and resilient agriculture, we propose that remote sensing should develop toward near-real time monitoring of certain traits on large scales under several environmental conditions (climate, soil etc.) as a global multi-site experiment. The current work on real-time observations (for example, special issue of MPDI remote sensing in 2021: “Near Real-Time (NRT) Agriculture Monitoring” https://www.mdpi.com/journal/remotesensing/special_issues/NRT_agriculture_monitoring) offers, on the one hand, the possibility to give timely management advice, which again could be optimized through joint research between the two communities. On the other hand, the remotely sensed information on plant traits and their actual condition could be directed back to field phenotyping experts to optimize the breeding lines with respect to certain environmental constraints. A similar concept for forests was proposed by Dungey et al. (2018).

We initiated a discussion between more than 130 experts from the remote sensing and plant phenotyping community in the context of a joint workshop (https://www.senseco.eu/working-groups/wg3-sensor-synergies/) of the COST action SENSECO “Optical synergies for spatiotemporal SENsing of Scalable ECOphysiological traits” (https://www.senseco.eu/) and the ESFRI plant phenotyping infrastructure EMPHASIS “European Infrastructure for Plant Phenotyping” (https://emphasis.plant-phenotyping.eu/). During the discussion, we identified the following key areas for future collaboration:

- transferring and harmonizing knowledge on protocols, methods, and data between the two communities;
- optimizing quantitative trait estimation by using new sensors, and integrating data from different spectral domains and spatial resolutions, preferably in real-time;
- linking existing and new modeling approaches and recent developments in artificial intelligence to bridge different observation scales through space and time.

## Data Exchange and Protocol Standardization

Plant phenotyping and remote sensing collect a large amount of data, including spectral observations and biochemical/biophysical plant traits. However, the exchange of these data requires the standardization of measurement protocols and harmonization of measurement procedures. Thus, a broad exchange of measuring concepts for plant trait assessments should be initiated, complemented by an open data policy allowing for the broader use and re-use of data (Fiorani and Schurr, 2013; Reynolds et al., 2019). In particular, plant phenotyping scientists are developing a large number of solutions to address a diversity of crops, traits, and treatments. Since there is no one-size-fits-all solution, existing hardware and software solutions often need to be adapted, even for the same traits of interests in different crops. This has led to both a “Phenotyping Dilemma” as stated by Rosenqvist et al. (2019) and the need for harmonization. This was addressed within COST action FA1306, “The quest for tolerant varieties – phenotyping at plant and cellular level” (Phenomen-All) and the EU-funded projects EPPN and EPPN2020 leading to the ESFRI research infrastructure entitled EMPHASIS. Plant phenotyping will never be able to measure all genotypes under all relevant conditions, thus, further integration of the community and the use and development of existing synergies, such as those between remote sensing and phenotyping, are key to achieving the required impact of improved plant production in times of climate change. It is therefore vital to develop FAIR data approaches (Wilkinson et al., 2016) that link the communities, and sharing phenotyping data (Danilevicz et al., 2021) will benefit plant and crop sciences at large.

In remote sensing, standards for metadata collection by scientists often mainly regard information on sensor performance or geolocation, while auxiliary data about vegetation is often limited to the traits in focus. But, plant status is only a small function of just one individual co-variable and requires additional information on the biotic and abiotic environment and genetic makeup of the plant, thus, such an approach may result in an oversimplified interpretation of the remote sensing signal (Galieni et al., 2021). In contrast, explicit geolocation is essential to link the signal to the field observation and needs to be considered in field phenotyping data collection.

The phenotyping community has developed the Minimum Information About a Plant Phenotyping Experiment (MIAPPE) as a bottom-up standard of metadata required to adequately describe a plant phenotyping experiment with well-defined data models and make the data reusable (Papoutsoglou et al., 2020). Such standards or developed recommendations and guidelines (Manfreda et al., 2018; Tmušić et al., 2020), like in the COST action CA16219 Harmonious (Harmonization of UAS techniques for agricultural and natural ecosystems monitoring, https://www.cost.eu/actions/CA16219/ and https://www.costharmonious.eu/), essentially represent a checklist of how to describe an experiment and could be adapted and extended by including the considerations of both communities to improve the interpretability, reusability, and transferability of data. This would also allow the exchange of data between the two communities and extrapolate results from one experiment to another. In particular, the phenotyping community could benefit from traits estimated within the landscape by remote sensing, while the remote sensing community could use data from the field phenotyping community to improve model development (c.f. section New Sensors for Quantitative Trait Estimation From the Plot and the Ecosystem Scale).

## New Sensors for Quantitative Trait Estimation From The Plot and The Ecosystem Scale

The availability of remote sensing data has increased significantly over the last decade. Satellites with a high temporal, spectral, and spatial resolution like the Sentinel-2 sensors or Hyperspectral Precursor and Application Mission (PRISMA) allow for new or improved agricultural applications. Moreover, the advent of nano-satellites further improves revisit times and spatial resolution of satellite systems. Developments in sensor technology, measurement procedures, and data correction workflows have matured UAVs to reliable quantitative remote sensing systems (Aasen et al., 2018) and initiated a new era in the remote sensing of crops (Zarco-Tejada, 2008; Herrmann and Berger, 2021). Today, a variety of (hyper) spectral, thermal, sun-induced fluorescence, and 3D/LiDAR instruments are available for UAVs. Consequently, they have also become a common tool for (high-throughput) field phenotyping (Yang et al., 2017) and have been proposed as a “game-changer in precision agriculture” (Maes and Steppe, 2019). Traits such as plant height/growth, pigments, canopy cover, and temperature, which are highly relevant for the vitality and performance of crops (Tattaris et al., 2016), can now be derived from UAV remote sensing data (Zarco-Tejada et al., 2012; Aasen and Bareth, 2018; Roth et al., 2018; Perich et al., 2020). Moreover, imaging spectroscopy from UAVs is able to capture data with a viewing geometry closer to the hemispherical-directional reflectance factors of satellite products (Aasen and Bolten, 2018) and thus may bridge the gap between field phenotyping experiments and landscape monitoring (Aasen and Herrera, (under review)). However, UAV flights need careful planning, consideration of regulations, and realistic estimation of manpower (Reynolds et al., 2019).

Another example where we expect that the increasing availability of UAV-based sensors will bridge the spatial gap for a better understanding of plant mechanisms is the assessment of photosynthesis (Quirós-Vargas et al., 2020). The analysis can be performed based on solar-induced fluorescence (SIF) in combination with established hyperspectral indices like the photochemical reflectance index (PRI) from remote sensing platforms (Rascher et al., 2015). SIF imaging provides information that could be used to identify genotypes that maintain a high level of photosynthetic activity under stress conditions. With the start of the FLEX mission (https://earth.esa.int/eogateway/missions/flex), global SIF data will be available to test stress resilience under varying environmental conditions, as stated in the introduction.

Furthermore, a combination of large-scale information with plant phenotyping, using high spatial and temporal trait measurement may help to identify different stress factors, in order to assess the stress stage and underlying mechanisms. For recent examples related to the identification of genotypes capable of tolerating biotic and abiotic stress, see recent reviews (Araus et al., 2018; Watt et al., 2020).

Thanks to the flexibility of remote sensing systems, the dynamic developments of plant traits can be assessed by standardized measurements with little effort. However, for more complex traits, such as the identification of biotic and abiotic crop stress, the selection of the most suitable sensor combination is challenging (Galieni et al., 2021; Berger et al., (in prep)). Nevertheless, remote sensing platforms such as UAVs and micro-satellites are overcoming traditional trade-offs between spatial, temporal, and spectral resolutions. Moreover, applying low-altitude and close-range remote sensing methods in combination with radiative transfer models (RTMs, c.f. section Bridging Observation Scales With Physically-Based Radiative Transfer Models and Machine Learning for Improved Trait Estimation) for field phenotyping allows several of the insights gained to be scaled to the ecosystem where they can be used for more precise field management (Velumani et al., 2021). In conclusion, the combined usage of different sensors may lead to an improved understanding of the actual carbon and water fluxes and to finding cultivars with higher resilience.

## Bridging Observation Scales With Physically-Based Radiative Transfer Models and Machine Learning For Improved Trait Estimation

In field phenotyping studies, parametric regression approaches are typically applied to link vegetation indices derived from multispectral data with plant traits. These models are easy to implement and require little expert knowledge. However, large datasets are needed for calibration and validation and still face the limited transferability of the established models to other crops and different environmental conditions. Moreover, especially when hyperspectral data are used, parametric regressions tend to under-exploit the comprehensive information content hidden in the contiguous spectral data (Verrelst et al., 2019). Therefore, remote sensing scientists have developed radiative transfer models (RTMs) simulating the interactions of the full optical wavelength range with leaves and canopies based on physical laws. Beyond the widely used one-dimensional (1-D) RTMs, which are suitable for homogenous canopy architectures, three-dimensional RTMs open up opportunities to analyze data generated by high-throughput field phenotyping experiments over row crops (Weiss et al., 2020). Thereby, remote sensing could provide spatio-temporal information on specific functional traits of interest. In combination with process modeling and data assimilation strategies, remote sensing could help to understand the processes in plants. One example in the context of sustainable agriculture is the estimation of nitrogen (N) use efficiency. Usually, N content is quantified indirectly from remote sensing data *via* the chlorophyll content (Chlingaryan et al., 2018) and very often still by a parametric regression based on vegetation indices. However, the quantification of the N content is challenging due to the unstable relation of N and chlorophyll, the very subtle spectral signals of proteins, and the dilution phenomenon, which often seems to be neglected in N concentration studies (Bossung et al. (under review)). Novel RTMs developed within the remote sensing community now allow the use of more flexible non-parametric models in the estimation N, which also take into account proteins and provide uncertainty estimations (Berger et al., 2020). Better estimating the plant N by combining information on plant physiology from plant phenotyping with new hyperspectral sensors giving a near-real-time estimation can provide input for optimized management strategies to reduce N applications and thus protect the water resources.

The integration of information from different spectral domains is complex and challenging. Models and tools have been developed to observe photochemistry and energy fluxes of the canopy. These include for example the SCOPE model where VIS/NIR data and fluorescence data are integrated (Van der Tol et al., 2009) but are still not perfect and are not widely used. Models and toolboxes are a big asset for the understanding of the interaction between vegetation and the environment. Further developments and interdisciplinary work are desirable to optimally combine the information from different sensors to fully describe the different traits, their interactions and the linked environmental triggers.

To obtain functional traits or to indirectly assess crop stress from the diversity of spectral data, a modeling framework should be defined. We propose the use of (shallow) machine learning (ML) regression algorithms combined with RTMs, such as SCOPE coupled with leaf optical properties models (Féret et al., 2021). Within these hybrid methods, training data sets are generated by the RTM and are then learned by the ML algorithm to build the specific retrieval model. Also, deep learning algorithms could be employed, in particular when a large number of different data sets are available and to better describe the highly non-linear relationship between remotely sensed signals and traits of interest. As an additional feature, the quality of training data can be enhanced by implementing active learning heuristics, which recently achieved outstanding results in the estimation of specific traits (Berger et al., 2021; Verrelst et al., 2021). All in all, these hybrid workflows may become a cornerstone for precision agriculture and an essential element for the development of new breeding strategies (Lammerts van Bueren and Struik, 2017).

## Conclusions and Future Perspectives

Plant phenotyping and remote sensing work with complementary measurements and concepts, but address the same challenge – namely, the quest for a more sustainable agriculture. Facilitating exchange between the two disciplines offers possibilities to trigger cross-fertilization: An improved understanding of target traits will allow the remote sensing community to develop more focused and precise tools. Likewise, establishing state-of-the-art remote sensing methods as plant phenotyping tools will allow improved understanding and modeling of crops in dynamic environments.

Working on harmonization and implementing open data standards allow the use and re-use of the data for a broader community. Further, bridging scales and concepts offers unique and promising approaches to address major long-term challenges identified both on national and large-scale levels: food security in changing climate conditions, resilient agriculture countering land degradation and erosion, sustaining biodiversity and ecosystem functions, and agro-ecological transition. UAVs are one important common tool that is bridging the technical gap between the two research domains. A huge challenge hereby is the careful (not only short-term price-driven) selection of sensors and appropriate spectral domains to obtain a maximum of information. Along with extensive trait measurements, remote sensing and crop growth models can be advanced, increasing our understanding of plant performance in a dynamic environment. This would result in remote sensing techniques becoming more reliable, increasing their usefulness for practical applications in precision farming.

We anticipate that there is a need to further stimulate cooperation and we advocate initiating projects and network activities between the remote sensing and plant phenotyping communities. Ultimately, this exchange has the potential to stimulate growth in both communities and their interconnection may lead to new developments toward more sustainable agriculture. These interactions may substantially contribute to the European strategic research agenda and the relevant topics are contributing to prominent parts of the EU Green Deal (“From farm to fork” and “EU Biodiversity Strategy”).

## Author Contributions

UR came up with the initial idea of bringing plant phenotyping and remote sensing scientists together. MM initiated the idea of a joint workshop and led the paper writing. MM, RP, KB, MS, SF, and UR jointly organized this workshop in February 2021, which marked the start of this article. HA completely revised the manuscript. All authors contributed to the writing and critical review of the article.

## Funding

This article is based upon work from COST Action CA17134 Optical synergies for spatiotemporal SENsing of Scalable ECOphysiological traits (SENSECO), supported by COST. It was supported by European Plant Phenotyping Network (EPPN2020: Grant Agreement 731013), from EMPHASIS-PREP (Grant Agreement: 739514), from EOSC-Life (Grant Agreement: 824087). KB was funded within the EnMAP scientific preparation program (DLR Space and BMWi, Grant Number 50EE1923).

## Conflict of Interest

The authors declare that the research was conducted in the absence of any commercial or financial relationships that could be construed as a potential conflict of interest.

## Publisher's Note

All claims expressed in this article are solely those of the authors and do not necessarily represent those of their affiliated organizations, or those of the publisher, the editors and the reviewers. Any product that may be evaluated in this article, or claim that may be made by its manufacturer, is not guaranteed or endorsed by the publisher.
